# A Point-Cloud Segmentation Network Based on SqueezeNet and Time Series for Plants

**DOI:** 10.3390/jimaging9120258

**Published:** 2023-11-23

**Authors:** Xingshuo Peng, Keyuan Wang, Zelin Zhang, Nan Geng, Zhiyi Zhang

**Affiliations:** College of Information Engineering, Northwest A&F University, Yangling 712100, China; pxs@nwafu.edu.cn (X.P.);

**Keywords:** semantic segmentation, deep learning, point cloud, downsample, plant phenotyping

## Abstract

The phenotyping of plant growth enriches our understanding of intricate genetic characteristics, paving the way for advancements in modern breeding and precision agriculture. Within the domain of phenotyping, segmenting 3D point clouds of plant organs is the basis of extracting plant phenotypic parameters. In this study, we introduce a novel method for point-cloud downsampling that adeptly mitigates the challenges posed by sample imbalances. In subsequent developments, we architect a deep learning framework founded on the principles of SqueezeNet for the segmentation of plant point clouds. In addition, we also use the time series as input variables, which effectively improves the segmentation accuracy of the network. Based on semantic segmentation, the MeanShift algorithm is employed to execute instance segmentation on the point-cloud data of crops. In semantic segmentation, the average Precision, Recall, F1-score, and IoU of maize reached 99.35%, 99.26%, 99.30%, and 98.61%, and the average Precision, Recall, F1-score, and IoU of tomato reached 97.98%, 97.92%, 97.95%, and 95.98%. In instance segmentation, the accuracy of maize and tomato reached 98.45% and 96.12%. This research holds the potential to advance the fields of plant phenotypic extraction, ideotype selection, and precision agriculture.

## 1. Introduction

Plant phenotyping is defined as all measurable in vitro manifestations of organisms, such as shape, structure, size, color, etc., that are determined by genotype and local environment [1]. A plant’s genes and growth environment, together, determine its phenotyping. The focus of this research is how to quantitatively understand the relationship between plant phenotypes and genotypes. This research may pave the way for a more comprehensive understanding of intricate genetic traits, ultimately serving modern breeding and precision agriculture [2,3,4]. Plant phenotyping data can also support automated crop picking [5,6,7]. Typically, plant phenotypic assessments center on the plant organs, including aspects like the characteristics of the leaves, stems, fruits, and root structures. With the largest surface coverage, leaves play a critical role in photosynthesis and respiration [8]. Thus, traits including the leaf area, length, width, and angle of inclination stand are pivotal phenotypic parameters [9]. Beyond the leaves, the stem serves as the plant’s foundational structure, linking it with other organs like flowers and fruits. Assessing stem phenotypes can also give insights into a plant’s stress levels [10,11].

The quick and accurate segmentation of plant organs is pivotal for plant phenotyping. Since the 1990s, there has been a growing interest in segmenting plant organs, with a significant focus on segmenting leaves to identify diseases. Techniques such as threshold-based segmentation [12], edge detection methods [13,14], region-growing techniques [15,16], clustering approaches [17], and their hybrids and advancements [18,19,20] are commonly used in 2D image-based phenotyping. Recent advances have seen convolutional neural networks (CNN) taking the forefront in tasks related to image classification and segmentation [21,22,23,24]. Deep learning frameworks have been utilized to differentiate between fruits and leaves in botanical images [23,25,26]. Nonetheless, it is worth noting that 2D phenotyping techniques are usually designed for less complex plants, such as rosette or monocot plants with fewer leaves.

3D models, compared to traditional images, offer not just color and texture insights but also depth information. This depth aspect effectively addresses problems arising from occlusions and overlaps, making it a cornerstone for precise phenotypic measurements. As affordable, high-precision 3D imaging technologies evolve, depth-based plant phenotyping tools have been burgeoning. Light Detection and Ranging (Lidar), known for its unparalleled precision in 3D imaging, is becoming the go-to method for the 3D reconstruction and phenotyping of various plants. It has been instrumental in studying tall trees [27,28], maize [29,30], cotton [31], and other economically valuable crops [32,33,34]. Additionally, 3D sensors that utilize Structured Light and Time-of-Flight (ToF) are gaining traction in 3D phenotyping due to their real-time processing capabilities [35]. Wang et al. [36,37] leveraged binocular stereovision for the 3D analysis of greenhouses and crops. Rose et al. [38,39] employed multi-view stereo (MVS) techniques for similar purposes. Miao et al. [40] introduced Label3DMaize, a tool designed for manually annotating point-cloud data. This tool streamlines the process of creating annotated 3D maize datasets, facilitating machine learning model training and evaluation.

While high-resolution 3D data present unparalleled accuracy in phenotyping, they concurrently raise intricate challenges. Foremost among these challenges are the effective disambiguation of individual plants from their growth environment and the subsequent partitioning of these plants into their constituent organs for phenotypic parameter extraction [41]. The unsupervised segmentation of leaves from 3D point clouds has emerged as a research area of significant intrigue. Paproki et al. [42] elevated the methodology for segmenting point cloud mesh, leading to the development of an adaptive hybrid segmentation model. This model exhibited adaptability to morphological variations across different cotton specimens, enabling discernment between leaves and stems. Duan et al. [43] segmented the plant point cloud into granular sections by employing the octree algorithm. Thereafter, they manually amalgamated these components into discrete organs, premised on spatial–topological associations. Itakura and Hosoi [44] advanced the adoption of the projection method integrated with attribute extension for leaf segmentation, and subsequently evaluated its segmentation accuracy across seedlings from six distinct plant species. The employment of the Difference of Normal (DoN) operator, as delineated by both [45] and [46], enabled the segmentation of leaves within point clouds for magnolia and maize, respectively. Furthermore, Zermas et al. [47] proffered a novel approach to segmenting overlapping maize leaves by leveraging node information inherent to the plant’s 3D architecture. However, it must be noted that the aforementioned point-cloud segmentation and phenotyping methodologies, despite their advancements, often exhibit limitations in their ability to universally segment a myriad of crop species characterized by varied leaf morphologies and canopy configurations [48,49]. The intricacies associated with parameter calibration in these segmentation approaches occasionally circumscribe their applicability. In a bid to surmount these challenges, Pan et al. [50] and Chebrolu et al. [51] innovatively employed spatiotemporal alignment. This strategy facilitated the linkage of plant organs during their growth phases, thereby enabling meticulous phenotypic growth surveillance.

At the forefront of 3D plant phenotyping, a critical pursuit is the development of a comprehensive 3D segmentation method that is applicable to plants at various growth stages. Recent strides in artificial intelligence have led to the rise of deep learning-based segmentation techniques that are tailored for unorganized and uneven point clouds, garnering attention in both academic research and the agricultural industry. Historically, researchers primarily delved into multiview Convolutional Neural Networks (CNNs) [52,53,54,55,56]. These studies aimed to bridge understandings of 3D data by reinforcing connections between 2D and 3D data through CNNs applied to images. However, challenges persisted, including difficulties in determining the angle and quantity of projection from a point cloud to a 2D image and the non-trivial reprojection from segmented 2D shapes back to the 3D space. Addressing these challenges, some researchers transitioned from 2D CNNs to voxel-based 3D CNNs [57,58,59,60,61]. In this approach, the point cloud undergoes subdivision into numerous voxels, and 3D convolutions facilitate direct segmentation on the point cloud. However, the computational demands associated with voxel-based 3D CNNs are considerable. In response to these challenges, there has been a notable shift towards more efficient and real-time segmentation models. Point-centric approaches, such as PointNet [62] and PointNet++ [63], operate directly on individual points, enabling simultaneous point-level classification and semantic segmentation. Subsequent enhancements to the PointNet-like framework have focused on optimizing and redesigning feature extraction modules [41]. For example, Masuda [64] successfully applied PointNet++ to semantically segment tomato plants in a greenhouse, extending its utility in estimating the leaf area index. Li et al. [65] designed a PointNet-like network for the semantic and instance segmentation of maize 3D data. Furthermore, recent advancements have introduced innovative approaches to address these challenges. The Similarity Group Proposal Network (SGPN) [66] and Graph Neural Networks (GNNs) [67,68,69,70,71] exemplify progress in this direction. SGPN employs a similarity matrix and group proposals, facilitating the simultaneous instance segmentation and semantic segmentation of point clouds. GNNs leverage the conversion of point clouds into connective graphs or polygon meshes to obtain information between adjacent nodes. The evolution of segmentation methodologies has progressed from multiview CNNs to voxel-based 3D CNNs and, most recently, to more lightweight and high-precision real-time segmentation models like PointNet and PointNet++. These advancements, along with innovations such as the SGPN and GNNs, underscore a concerted effort to address the unique challenges associated with 3D plant phenotyping at different growth stages, emphasizing the need for improved efficiency and real-time capabilities in segmentation networks.

A well-designed downsampling algorithm can effectively enhance the segmentation accuracy of training models. Typically, the downsampling process should ensure a reduction in data density while retaining key shapes, structures, and features in the point cloud. Commonly used point-cloud downsampling algorithms include Random-Sampling (RS) [72], Uniformly Voxelized Sampling (UVS) [73], and 3D Edge-Preserving Sampling (3DEPS) [74], among others. Dawei Li et al. [47] introduced Voxelized Farthest Point Sampling (VFPS), a novel point-cloud downsampling strategy, to prepare plant datasets for training deep neural networks. This method significantly improves the segmentation accuracy of plant point clouds. However, compared to industrial products, the study of plant point clouds faces challenges due to the irregularity of their 3D structures. Therefore, commonly used point-cloud downsampling algorithms may not be well-suited for applications in crop point-cloud segmentation. 

Furthermore, to further enhance the segmentation accuracy of point clouds, researchers are exploring the inclusion of variables beyond positional information during the segmentation process, such as the reflectance intensity [75], point cloud color [76], and more. Given that the 3D structures of plants undergo significant changes over time, incorporating time variables as inputs for training models may effectively improve their segmentation accuracy. Currently, there is limited research in this area.

To summarize, deep learning has been recognized as a promising methodology for achieving precise organ segmentation and intricate phenotypic trait elucidation within plant point clouds [77]. However, several issues remain unsolved—(i) because of the huge disparity in the number of points between the different organs of the crop, there is no standardized downsampling methodology tailored expressly for point clouds in preparation for deep learning applications; (ii) it is still challenging to design lightweight and high-precision real-time network segmentation models; and (iii) because the structure of crops will change significantly during their growth and development, a segmentation network that incorporates temporal mediating is cogent.

In response to the aforementioned challenges, we architect a sophisticated deep learning framework for the plant-organ semantic segmentation of plant phenotypes in manually labeled point-cloud datasets. The network achieves ideal segmentation results on tomatoes and maize. Our contributions include three aspects:

(i) We put forth an innovative point-cloud downsampling technique that adeptly amalgamates the merits of voxelization-based sampling (VBS) and furthest point sampling (FPS). This method proficiently addresses the sample imbalance quandary. This technique enhanced the stability of point-cloud data via random initialization during the sampling process. Ablation studies underscore that this downsampling approach markedly enhances the precision of crop-organ semantic segmentation;
(ii) We also take time series as input variables. The structure of some plants will change dynamically as they grow. Taking time series together with other features as input variables can make full use of the information of these features and provide a more comprehensive input signal to improve the segmentation performance;
(iii) We introduce a robust deep architecture for plant-point-cloud segmentation. Upon training with a dataset refined through our downsampling technique, this model proficiently accomplishes the semantic segmentation of both stem and leaf categories. Compared with several mainstream deep learning networks such as PointNet++ [63], DGCNN [71], and JSNet [78], our network emerges superior, delivering unparalleled segmentation outcomes both in qualitative and quantitative measures. Furthermore, we conduct meticulous ablation studies on the network’s constituent modules, reaffirming their instrumental efficacy.

## 2. Materials and Methods

### 2.1. Data Preparation

**Figure 1:**
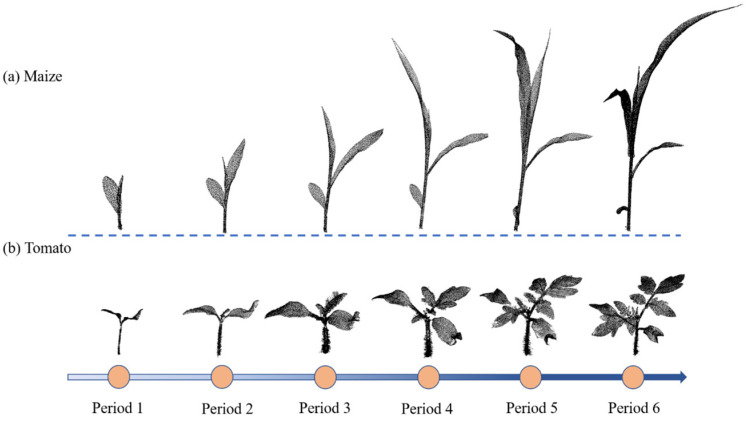
Two examples of crop-point-cloud dataset. (**a**) Point cloud of the same maize plant for six consecutive growth periods; (**b**) point cloud of the same tomato plant for six consecutive growth periods.

The dataset used in this paper comes from public data on the internet. [79]. The dataset is composed of data for two crops including maize and tomato. The scanning device was a Perceptron Scan Works V5 laser triangulation scanner (Perceptron Inc., Plymouth, MI, USA), and each crop underwent multiple scanning sessions spanning three weeks. Two crops at different growth stages are shown in Figure 1. The dataset’s scanning discrepancy is meticulously regulated to remain within a 0.1 mm threshold. We selected 222 individual point clouds in the dataset, including 84 maize point clouds (about 90 million points) and 138 tomato point clouds (about 350 million points). Acquisition time is recorded for each set of point-cloud data.

The points from Pheno4D are categorized as “ground,” “stem,” or “leaf,” where each point on the same leaf is assigned a unique label, distinguishing it from other leaves on the same plant. Since our network primarily focuses on segmenting stems and leaves in plant-organ point clouds, we utilized CloudCompare v2.12 software to remove the points labeled as “ground.” As a result, all plant point-cloud labels can be classified into two major categories: “stem” or “leaf,” serving the purpose of semantic segmentation. Stems have a unique semantic label, and each individual leaf, in addition to the semantic label, is assigned a unique instance label. To better train and test the network, the dataset necessitates bifurcation into a training set and a test set. In this study, we strategically partition the original dataset into training and test sets at a proportion of 2:1.

### 2.2. Point Cloud Sampling

An unbalanced number of different types of point clouds will cause problems such as overfitting and low segmentation accuracy. Therefore, while the point cloud sampling method can reduce the number of points and retain key features, it also needs to be able to balance the number of samples of each category in the training dataset. Presently, two sampling methodologies are predominantly adopted: Voxel-Based Sampling (VBS) [80] and Farthest Point Sampling (FPS) [71]. 

**Figure 2:**
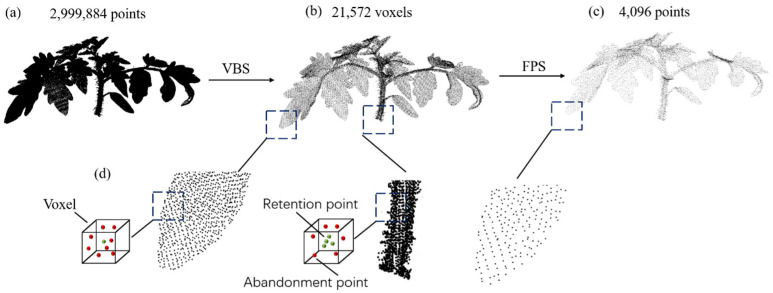
Schematic illustration of the point cloud sampling method. (**a**) Raw point cloud. (**b**) Point cloud after VBS. (**c**) Point cloud after FPS. (**d**) Specific details for improving VBS.

We analyze the advantages and disadvantages of FPS and VBS and propose a new sampling method. Our sampling approach combines VBS and FPS, with a primary focus on enhancing the VBS sampling process. In the traditional VBS sampling procedure, each voxel selects only one point, potentially leading to uneven sampling, especially in regions with varying densities. This can result in an uneven distribution of point-cloud densities, impacting the comprehensive representation of the original point cloud. The density distribution in plant point clouds, such as tomato plants, tends to be uneven. The leaf areas of tomatoes, with a larger surface area, exhibit higher point-cloud density but contain limited detail. In contrast, the stems have complex structures and include more detailed information. If the same sampling parameters are applied, it could lead to redundancy in leaf point clouds while losing structural details in the stems. To address this issue, we employ weighted adjustment during the sampling process. Each voxel is assigned a unique weight to increase the likelihood of selecting voxels from regions with lower point-cloud densities. These weights can be adjusted based on factors like the number of points or other voxel-specific features. This adaptive sampling approach aims to mitigate the uneven distribution of point-cloud densities. In summary, our combined VBS and FPS sampling method, with a focus on refined VBS sampling and weighted adjustments, addresses the challenges posed by uneven point-cloud density distributions, especially in plant structures like tomato plants. This methodology unfolds in three stages, as illustrated in Figure 2. The leftmost point-cloud depiction (a) represents the raw tomato point cloud, encompassing a total of 2,999,884 points. Subsequently, VBS is employed on the raw point cloud, resulting in a new point cloud comprising 21,572 voxels, as depicted in (b). In this paper, we propose a sampling method with weighted adjustments based on Voxel-Based Sampling (VBS). Initially, guided by prior knowledge, we labelled point clouds with larger densities and fewer details as Type A. In this context, leaf point clouds are specifically identified as Type A. Conversely, point clouds with more detailed information are categorized as Type B, with stem point clouds being marked as Type B in this study. The proportion between Type A and Type B is denoted as ‘*n*’, and, in this paper, the value of ‘*n*’ is set to 3. In cases where a voxel contains Type A points, similar to the traditional VBS approach, only the point closest to the voxel’s centroid is retained. In voxels containing exclusively Type B points, the closest ‘n’ points to the voxel’s centroid are retained. If the number of Type B points within a voxel is less than ‘*n*,’ all such points are retained. This sampling method not only preserves structural information, allowing the downsampled point cloud to reflect the fundamental shape of the original point cloud, but also mitigates the issue of uneven sampling. This detailed procedure is elucidated in (d). Finally, FPS is applied to generate the final result (c) with exactly 4096 points.

This sampling method has the advantages of both VBS and FPS. For each point cloud, we use randomly selected initial points to repeatedly downsample 300 times to enhance robustness. The stochastic nature of data augmentation arises from the variability in the FPS initial iteration points after voxelization. This sampling method enables replicable experiments, improving the reproducibility of the study through multiple samplings of the same plant point cloud. Introducing a controlled degree of randomness in the sampling process enhances the model’s generalization ability. Furthermore, this acquisition method captures authentic plant characteristics in the generated samples, preventing the loss of crucial information that could impact the model’s performance. The meticulous control of point-cloud density during sampling ensures that the resultant samples contain sufficient information for an accurate representation of plant shape and structure. For the test set, we downsampled the point cloud to *N* = 4096 through random sampling and repeated random sampling 300 times for each point cloud.

### 2.3. Network Architecture

**Figure 3:**
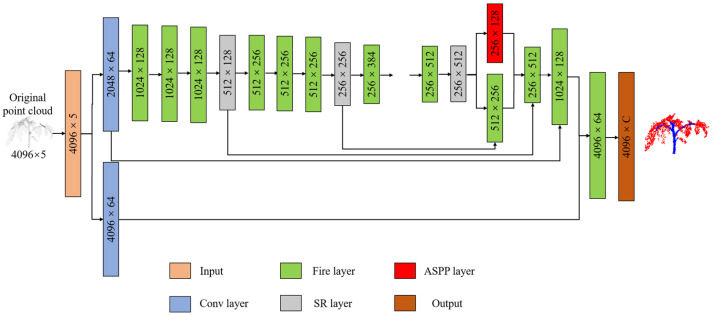
Deep learning network structure. Input represents the input matrix, Conv represents the convolutional layer, ASPP represents the Atrous Spatial Pyramid Pooling layer, SR represents the squeeze reweighting layer, and Output represents the output matrix.

The deep learning network designed in this paper has three main functional layers: (1) Fire Layer, (2) squeeze reweighting (SR) layer, and (3) Atrous Spatial Pyramid Pooling (ASPP) layer. As illustrated in Figure 3, the network structure is delineated. 3D point clouds are conventionally represented as sets of Cartesian coordinates (*x*, *y*, *z*). Solely relying on this three-dimensional coordinate system poses challenges to effectively discerning the nuances of point-cloud features. Therefore, many scholars try to add additional parameters, including point color (R, G, B) and reflection intensity. To better expand the dimensional characteristics of the point cloud, this paper takes the distance d = $\sqrt{x^{2}+y^{2}+z^{2}}$ from the crop coordinates to the origin as the input of the fourth dimension. Entering distance as an independent dimension allows the model to converge as quickly as possible. Compared with common industrial products, the three-dimensional structures of crops will change during growth, and the morphological structures of crops in different periods may vary vastly. Therefore, it is difficult to segment crop organs at different growth stages. To reduce the impact of crop growth and development on model accuracy, we take the time series as the input of the fifth dimension. 

In this study, we focus on the point-cloud analysis of crops, with the time series representing the growth cycle of the crops. The growth cycle refers to the time required for a specific crop to go from seeding to harvesting. The format of the time series in this study is in the form of timestamps. Timestamps represent the time point corresponding to each data point. Timestamps can use various time units, such as years, months, days, hours, minutes, or seconds, depending on the data collection frequency and application context. Timestamps can be in the format of date and time or numerical values. In this study, timestamps are in the daily format and represented as numerical values. The timestamp for each plant point cloud is the difference in days between the date of collecting the plant point cloud and the seeding date. Similar to three-dimensional coordinate information, timestamps are treated as independent input variables during training. During each batch of training, sample crops in different growth stages are selected for training. The minimum batch sample size selected in this paper is 4096, such that the training input matrix is 5 × 4096. The point clouds to be trained are randomly sorted and sent to the network in batches for training.

#### 2.3.1. Fire Layer

“SqueezeNet” epitomizes a streamlined convolutional neural network architecture, meticulously crafted to curtail the model’s parameter count and computational intricacy without compromising its precision [58]. It uses a special hierarchical structure called “Fire Module”, hence the name “Fire Layer”. SqueezeNet has the advantages of a small number of parameters and calculations, high efficiency, accuracy, and scalability, and a wide application range. Because of these advantages, SqueezeNet has been widely used in scenarios with limited computing resources and real-time requirements, including mobile terminals, autonomous driving, and intelligent monitoring. The Fire Layer is a core component in SqueezeNet for extracting and learning semantic segmentation-based stations. Its design is inspired by the Inception module and the bottleneck structure and aims to reduce the size of the model by reducing the number of input channels.

A Fire Layer consists of two parts: the squeeze part and the expand part. The “squeezing” component employs a 1 × 1 convolutional layer to condense the channel count of the input data, subsequently diminishing the model’s parameter tally. This strategic reduction alleviates computational burdens, bolstering the model’s efficacy. After this, the “expanding” phase bifurcates into two distinct subcomponents: a 1 × 1 convolutional layer (termed “squeeze expansion”) and a 3 × 3 convolutional layer (denoted as “expand expansion”). These dual subparts are engineered to instigate non-linear transformations and amplify feature dimensionality.

**Figure 4:**
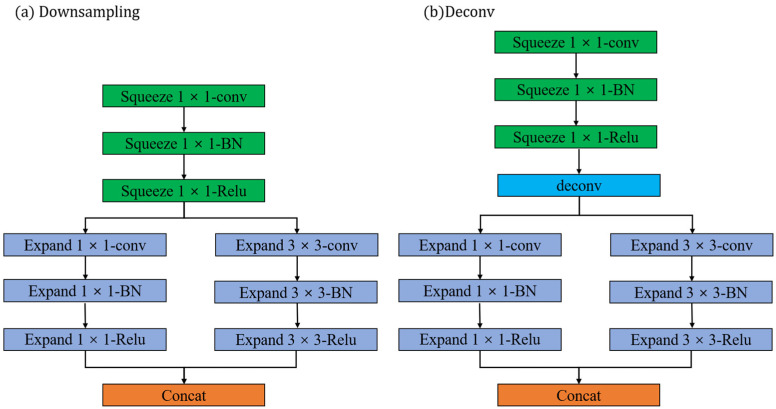
The structure of the Fire Layer. (**a**) is the fire model in the downsampling process, and (**b**) is the Deconv model in the upsampling process. conv represents the convolution operation, BN represents the normalization operation, Relu represents the activation function, squeeze represents the extrusion layer, expand represents the expansion layer, concat represents the concatenation of the matrix, and Deconv represents the transposed convolution layer.

The Fire Layer aims to reduce the model size while maintaining efficient feature representation by compressing and expanding feature channels. This design enables the network to operate in resource-constrained environments while providing high accuracy. By introducing the Fire Layer, the network achieves performance comparable to traditional deep neural networks with fewer parameters and calculations. This makes the network an effective choice for real-time classification and object detection in resource-constrained environments. The model used in this article has a total of 13 Fire Layers, of which the first 8 are downsampling layers, as shown in Figure 4a, and the last 4 are Deconv layers, as shown in Figure 4b.

#### 2.3.2. ASPP Layer

ASPP is a feature extraction method for semantic segmentation tasks, designed to capture information at different scales [81]. In conventional convolutional neural network architectures, pooling layers serve a dual purpose: they not only diminish the dimensions of feature maps but also amalgamate contextual data, invariably at the cost of forgoing spatial or location-specific information. However, in point-cloud segmentation tasks, location information is very important for accurate semantic segmentation. In the realm of point-cloud segmentation tasks, where the preservation of location information stands as a pivotal factor for achieving precise semantic segmentation, this paper employs the ASPP algorithm to tackle the inherent challenges posed by conventional pooling layers.

ASPP is a feature integration technique that addresses the issue of spatial information in point-cloud segmentation tasks through multi-scale dilated convolutions. ASPP employs parallel dilated convolutional layers, each with different dilation rates, to capture contextual information at various scales. This approach enables the network to maintain location information while comprehensively understanding structures of different sizes and scales within the point cloud, thereby enhancing the accuracy of semantic segmentation. ASPP effectively addresses the impact of sacrificed location information in point-cloud segmentation tasks due to pooling layers. ASPP expands the receptive field information by introducing dilation in the convolution operation at different dilation rates. ASPP consists of multiple parallel convolutional layers, each with a different dilation rate. 

**Figure 5:**
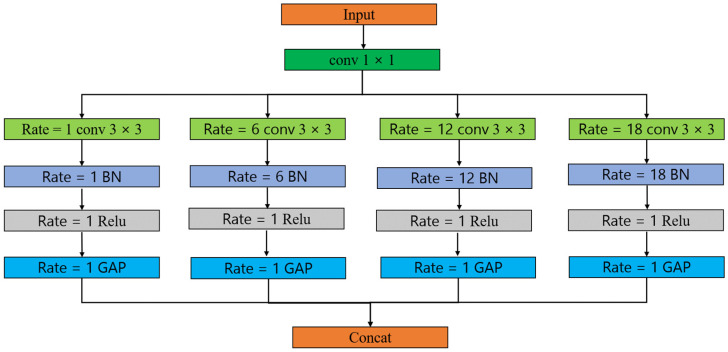
The structure of the ASPP layer. The rate represents the expansion rate, conv represents the convolution operation, BN represents the normalization operation, Relu represents the activation function, GAP represents the pooling operation, and concat represents the concatenation of the matrix.

In ASPP, each parallel convolutional layer uses global average pooling to preserve global context information. Then, the pooled features are concatenated or superimposed with the result of the convolution operation at the corresponding dilation rate to form the final ASPP feature representation. The structure of ASPP can be adjusted according to the requirements of specific tasks, including choosing different dilation rates and convolution kernel sizes. Its goal is to improve the performance of semantic segmentation through multi-scale feature-receptive fields and provide richer context information while preserving position information. The specific structure is shown in Figure 5.

In this structure, ASPP includes an initial 1 × 1 convolutional layer to reduce the number of channels of features. Then, four convolution operations with different dilation rates and corresponding pooling operations are applied in parallel. The convolution operation and pooling operation of each branch help to capture the receptive field information of different scales. Finally, the results of each branch are stitched together and the final ASPP features are output.

#### 2.3.3. SR Layer

**Figure 6:**
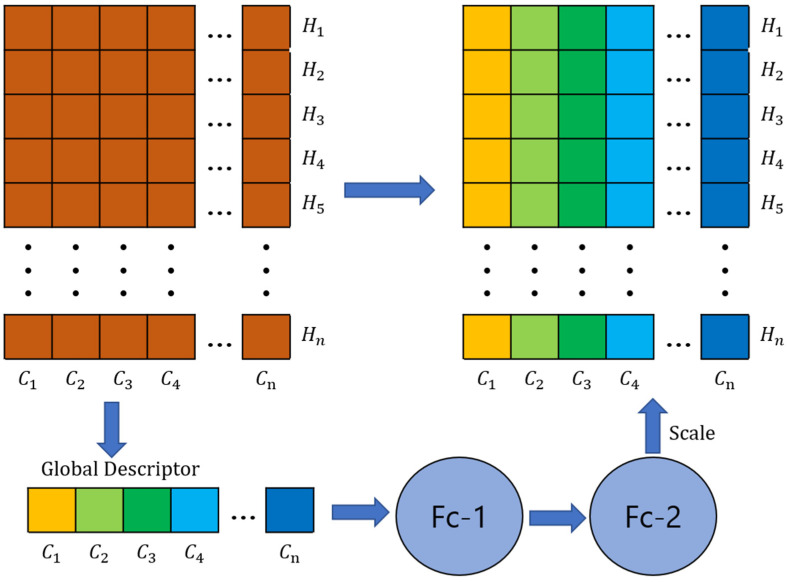
The structure of the SP layer. Fc represents the fully connected layer.

The SR layer is a module for enhancing feature representation. It is mainly used to increase the expressive power of the network and to learn the importance of features adaptively. The name of this layer comes from its two main operations: Squeeze and Reweighting. The structure of the SP layer is shown in Figure 6.

Initially, a global average pooling operation is executed on the input features, effectively condensing them into a singular vector. Assume the dimensions of the input matrix to be (*C*, *H*), where *C* denotes the channel count and *H* represents the total number of points. By undertaking average pooling on the feature values spanning each channel, the feature dimensionality undergoes a reduction from (*C*, *H*) to (*C*, 1). Post this compression, the resulting matrix *X* is defined as:

(1) $$X_{n}=\frac{1}{H}\sum_{i=1}^{H}p_{n}\left(i\right),n\in C,i\in H$$

This captures global information and reduces the dimensionality of features. Next, after obtaining channels of shape 1 × *C* (as shown in Figure 6), we use two fully connected layers to generate channel dependencies (called Scale in Figure 6). To reweight channel correlations, *Y* is formulated as yielding a weight vector representing the importance of each channel.

(2) $$Y_{n}=X_{n}\times {Scale}_{n},\;n\in C$$

Finally, the generated weight vector is applied to the original features, and the features are reweighted by multiplying the features of each channel by the corresponding weight value. This allows the network to learn which channels are more important to the task and focus more attention on these important features. Learning adaptive feature weights can improve the expressive power and performance of the model.

### 2.4. Loss Functions

The Cross-Entropy Loss function is ubiquitously employed in classification endeavors to quantify the disparity between the probability distribution of the model’s output and the ground-truth label. In this manuscript, we opt for the point-wise cross-entropy loss function to juxtapose the forecasted semantic categorization against the authentic semantic label. The cross-entropy between the anticipated category and the genuine category is computed, wherein the softmax function is utilized to transmute the projected category into a probability distribution, subsequently aligning it with the true label. The formulation of the loss function is articulated as follows:

(3) $$L_{loss}=-\frac{1}{N}\sum_{i=1}^{N}\sum_{c=1}^{C}y_{i,c}\log\left(p_{i,c}\right)$$

wherein *N* signifies the cumulative count of points in the point cloud, and *C* denotes the number of distinct semantic categories. The term *y_i,c_* designates the actual category label for the *i*-th point, while *p_i,c_* encapsulates the projected category probability distribution for said *i*-th point.

### 2.5. Instance Segmentation

To obtain the final leaf point cloud, further instance segmentation is required. The crop instance segmentation object studied in this paper is the leaf. Considering that there is a certain distance between different leaves and that the density distribution is quite different, this paper adopts the MeanShift algorithm to further divide the crops. The quintessence of the MeanShift algorithm lies in discerning clusters within data by perpetually gravitating data points toward regions of heightened density. Notably, the algorithm absolves the need to pre-specify the cluster count and operates without any presupposed constraints on the cluster’s morphology.

The MeanShift algorithm is based on kernel density estimation, which estimates the density distribution of data by calculating the density of data points around each data point. The Gaussian kernel function is customarily employed to ascertain the similarity between data entities. For a pair of data points, denoted as *x* and *y*, the computation of the Gaussian kernel function is delineated as follows:

(4) $$K(x,y)=\frac{exp(-||x-y||2}{\left(2\times h2\right)}$$

Among them, ||*x* − *y*|| represents the Euclidean distance, and h is the kernel bandwidth.

The algorithm finds clusters in the data by iteratively shifting the data points. For each data point, it is moved to an area of higher density by computing a weighted average of its surrounding data points. The movement continues until the data points no longer move significantly or until a stop condition is reached. For a given data point *x*, the mean shift vector is calculated as follows:

(5) $$MeanShift(x)=\frac{\Sigma (K(x,xi)*xi)}{\Sigma (K(x,xi))}-x$$

Among them, *xi* is other data points in the data set, and *K*(*x*, *xi*) is the similarity between *x* and *xi*, calculated by the kernel function.

In each iteration, the MeanShift algorithm updates the size of the window according to the position of the current data point. Finally, the data points are assigned to the nearest cluster center to form the clustering result.

### 2.6. Evaluation Metrics

To rigorously assess and juxtapose the performance of the model on point-cloud datasets is imperative, both as a benchmarking tool and to illuminate avenues for model and algorithmic enhancement. This paper selects the following five indicators as the evaluation indicators for point-cloud semantic segmentation: *Precision*, *Recall*, *F*1-*score*, Intersection over Union (*IoU*), and *Accuracy*.

(6) $$Precision=\frac{TP}{TP+FP}$$

(7) $$Recall=\frac{TP}{TP+FN}$$

(8) $$F1-score=2\times \frac{Precision\times Recall}{Precision+Recall}$$

(9) $$IoU=\frac{TP}{TP+FP+FN}$$

(10) $$Accuracy=\frac{TP+TN}{TP+TN+FP+FN}$$

In the above formula, *TP* (True Positive) indicates the number of samples that are truly positive examples that are correctly predicted as positive examples, and *FP* (False Positive) indicates the number of negative examples that are incorrectly predicted as positive examples. *TN* (True Negative) indicates the number of samples that are truly negative examples that are correctly predicted as negative examples, and *FN* (False Negative) indicates the number of positive examples that are incorrectly predicted as negative examples. The value range of the above indicators is between 0 and 1, and the higher the value, the higher the prediction accuracy of the model.

## 3. Results and Discussion

### 3.1. Training Details

**Figure 7:**
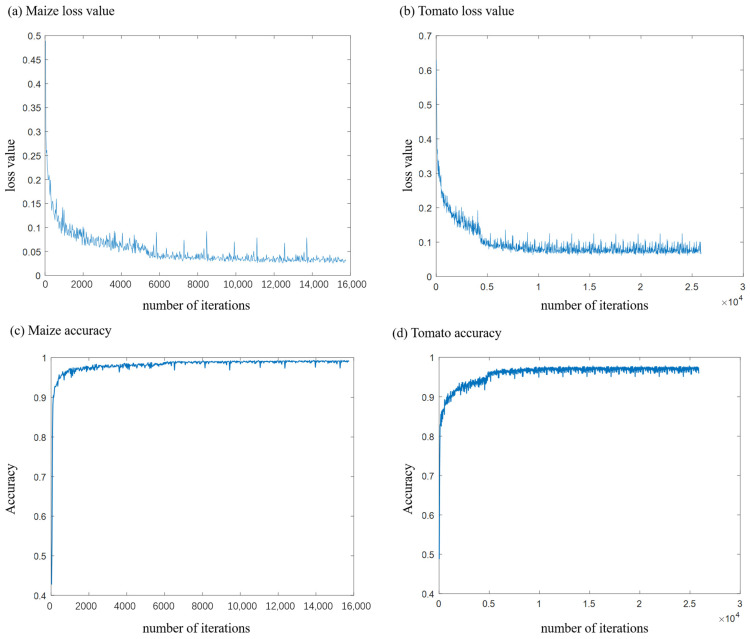
Variations in the loss function throughout neural network training (**a**) illustrate the evolution of the loss function during maize training, (**b**) portray the loss fluctuation during tomato training, (**c**) illustrate the evolution of accuracy during maize training, and (**d**) portray the fluctuation of accuracy during tomato training.

Our computational infrastructure is anchored by an Intel Core i7-13700K CPU, boasting 12 cores and 20 threads, and complemented with 512 GB of RAM and an NVIDIA RTX 3060 GPU. We employ the PyTorch framework for deep learning. Throughout the training phase, we consistently employ a batch size of 64. The learning rate is initialized at 0.005, undergoing 50% decrements every 10 epochs. The optimization of our network leverages the Adam Solver, with the momentum calibrated to 0.9. The training is capped at 60 epochs, and the model iteration exhibiting the minimal validation loss is earmarked as the optimal model. This manuscript also chronicles the trajectory of the loss function during the maize and tomato training sessions, as depicted in Figure 7. A swift convergence is evident across all loss functions.

Among them, the number of point clouds in the maize training set is 56, each point cloud is sampled 300 times, making a total of 56 × 300 = 16,800 training samples, and the batch size is 64. Upon termination of the training at 60 rounds, the *x*-axis is positioned at 16,800 / 64 × 60 = 15,750 ends. The number of tomato training sets is 92 point clouds, and each point cloud is sampled 300 times, making a total of 92 × 300 = 27,600 training samples. The batch size is 64. Upon termination of the training at 60 rounds, the *x*-axis is positioned at 27,600 / 64 × 60 = 25,875 ends.

### 3.2. Semantic Segmentation Results

**Figure 8:**
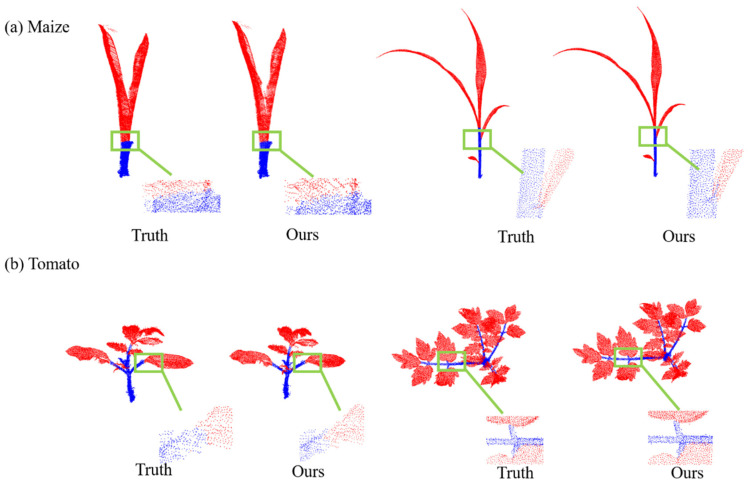
Point-cloud semantic segmentation results. (**a**) Maize Point-cloud semantic segmentation results. (**b**) Tomato Point-cloud semantic segmentation results.

Figure 8 shows the qualitative results of the network for the semantic segmentation of two crops, maize and tomato. To show the segmentation effect of the network more intuitively, we selected crop plants at different growth stages as examples. From the presented figure, it is evident that a significant majority of the points are segmented accurately. Nonetheless, a few points at the interface between the stem and the leaf might exhibit potential misclassification.

Table 1 presents the outcomes of the network’s semantic segmentation performance on the test dataset, on which all metrics achieved 95.0% or more, showing superior semantic segmentation performance. It is easy to check that the stem and leaf segmentation results are balanced, and are not affected by the imbalance of samples. Although the training samples of maize are fewer than those of tomato, the overall segmentation accuracy of the maize is better than that of the tomato point cloud. This is probably because of the relatively simple structure and obvious three-dimensional characteristics of maize itself.

### 3.3. Instance Segmentation Results

**Figure 9:**
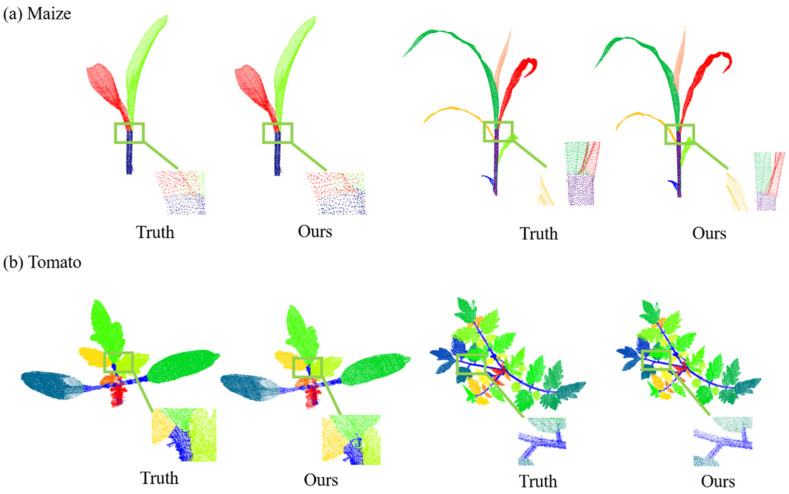
Point-cloud instance segmentation results. (**a**) Maize point-cloud instance segmentation results. (**b**) Tomato point-cloud instance segmentation results.

Figure 9 illustrates the qualitative outcomes of employing the MeanShift algorithm for the instance segmentation of both the maize and tomatoes, including four representative point clouds of different growth stages. Although the leaf structures of the two crops were quite different, the characteristics of the density distribution were similar. Therefore, the MeanShift algorithm based on the density distribution shows good performance for leaf instance segmentation.

Table 2 presents the quantitative results obtained from the application of the MeanShift algorithm for the instance segmentation of the maize and tomato crops. The instance segmentation accuracy exceeds 95%, indicating a superior performance of the proposed method. This achievement can be attributed to the algorithm’s high sample precision and the limited presence of noise points, which could potentially cause interference between distinct leaves.

The MeanShift algorithm operates by leveraging the peak probability density of data points in the feature space, thereby identifying regions with higher density within the dataset. However, it is crucial to acknowledge that an increased number of noise points may lead to a higher error rate within the predictions. In such scenarios, there is a possibility that the algorithm might misclassify two leaves and assign them to the same category. Consequently, it becomes imperative to mitigate the impact of noise and ensure the dataset’s quality to enhance the accuracy of the MeanShift algorithm in the context of instance segmentation tasks. The dataset for this study comes from a public source which was collected indoors with high precision and minimal noise. This minimizes the impact of noise on the MeanShift algorithm. Our semantic segmentation accuracy is noteworthy, providing a strong foundation for MeanShift’s instance segmentation. During the computations, we carefully tuned the bandwidth parameters to effectively smooth out noise.

Utilizing this high-precision indoor dataset and prioritizing semantic segmentation accuracy, we have established MeanShift’s reliability. Proper parameter tuning during computations ensures effective noise reduction, contributing to the robustness and credibility of our findings.

### 3.4. Comparison with Other Methods

Here, we conduct a comprehensive comparative analysis between our proposed network and several prominent point-cloud segmentation networks using the same plant dataset. The networks under consideration are PointNet [62], PointNet++ [63], JSNet [78], and DGCNN [71].

To ensure a rigorous and fair comparison, we adhere to the network parameter configurations recommended in the original papers. Moreover, in pursuit of optimal comparability across the networks, we uniformly train each model, employing an identical set of input variables and semantic labels. The input point clouds for each network are the same and have undergone the downsampling method proposed in this paper.

It is essential to emphasize that, for this study, we intentionally exclude time-series data as input variables. By employing this meticulous approach to training and evaluation, we can effectively ascertain and scrutinize the semantic segmentation capabilities of our proposed network relative to other state-of-the-art models in the field of point-cloud analysis.

Table 3 presents a comprehensive quantitative comparison of the five considered networks in terms of their performance on the semantic segmentation task. Notably, our proposed network obtains superior results on all evaluated scenarios, surpassing other methods in all four quantitative metrics by clear margins.

**Figure 10:**
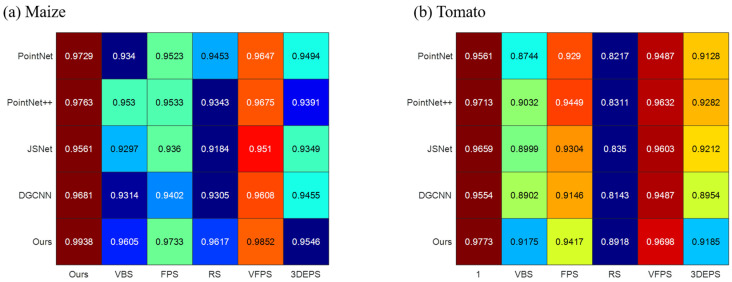
Comparison results of sampling methods. (**a**) The impact of different sampling methods on the segmentation accuracy of maize point clouds. (**b**) The impact of different sampling methods on the segmentation accuracy of Tomato point clouds.

To thoroughly evaluate the proposed downsampling method, we conducted a comprehensive comparison with VBS, FPS, RS, VFPS, and 3DEPS. As depicted in Figure 10, we integrated all sampling methods into various networks (PointNet, PointNet++, JSNet, DGCNN, and ours) for a comprehensive comparison. Through the comparison, it becomes evident that our proposed downsampling method yields the highest improvement in segmentation results. Additionally, the sampling principle of VFPS, which bears similarity to our proposed method, also demonstrates a notable enhancement in segmentation outcomes. On the other hand, random sampling, despite its computational efficiency, performs the least favorably. The inherent structure preservation deficiency of random sampling may result in uneven representations of different regions, potentially leading to the loss of critical details in densely populated areas or structures.

Each sampling method exhibits satisfactory performance in maize segmentation results, which can be attributed to the relatively simple structure of maize, especially in the case of its stem. However, the structure of tomatoes is more intricate, particularly in the case of tomato stems. Consequently, sampling methods that fail to effectively preserve the inherent structure of point clouds can severely compromise the segmentation accuracy of tomatoes.

It is noteworthy that the choice of sampling method significantly influences the performance of each segmentation network. A well-designed sampling approach can effectively enhance segmentation results.

### 3.5. Ablation Study

We use ablation experiments to analyze the impact of different modules and methods on segmentation results. This encompasses the downsampling module, the ASPP, and the time series. The outcomes of these ablation tests pertaining to semantic segmentation are tabulated in Table 4. An analysis of Table 4 reveals that the time series marginally influences the precision of point-cloud segmentation, particularly for maize. However, a more pronounced impact is observable in the segmentation accuracy of tomatoes. This may be due to the strong self-similarity of the structure of maize, which will not change its structural characteristics during growth. The additional time series may even cause a decrease in the segmentation accuracy of the maize point cloud. We believe that the reason for this phenomenon lies in the complex growth structure of and morphological changes in tomatoes. Due to the branching and structural alterations that tomatoes undergo during growth, time-series information is crucial for capturing these changes accurately. Therefore, compared to plants with simpler growth structures like maize, tomatoes exhibit greater sensitivity to changes in the time series, resulting in a more significant impact on the segmentation accuracy.

For crops like maize, where the structural changes during growth and development are not significant, omitting time-series data might prevent a decline in point-cloud segmentation precision. In contrast, for crops like tomatoes, which undergo substantial structural changes as they grow, introducing additional time series during periods of pronounced structural variation could enhance the segmentation accuracy. By strategically incorporating extra time-series data during key growth stages when structural changes are notable, we can further improve the precision of segmentation. This approach acknowledges crop-specific dynamics and tailors the use of time-series information accordingly to optimize segmentation outcomes.

Therefore, using time series as an input variable can improve the point-cloud segmentation accuracy for tomato. In summary, for crops whose structure changes dynamically during the growth and development process, using time series as an input variable can effectively improve the segmentation accuracy.

It can be seen from Table 4 that the unbalanced downsampling method proposed in this paper can improve the accuracy of point-cloud segmentation. The difference in evaluation metrics between stems and leaves is the largest without the downsampling module. Especially for tomato plants, the number of leaf point clouds is much larger than the number of stems. This phenomenon caused the overfitting of the model, and a large number of stem points were misclassified as leaf points. Consequently, the downsampling methodology introduced in this study adeptly addresses the sample imbalance predicament, culminating in enhanced accuracy in point-cloud segmentation.

The ASPP module can also effectively improve the segmentation accuracy of point clouds. This module improves the segmentation accuracy of tomato point-clouds more significantly but does not significantly improve maize’s segmentation accuracy. This may be due to the complex leaf distribution and structure of tomatoes. By incorporating the ASPP module, the network can effectively capture global and local contextual information, enabling it to make more informed decisions during semantic segmentation. The module helps the network to consider a wider range of object scales and contextual cues, leading to an improved segmentation performance and an enhanced semantic understanding at a fine-grained level. Overall, the ASPP module contributes to the network’s ability to better perceive and interpret the semantics of a point cloud, resulting in more accurate and detailed segmentation results.

## 4. Conclusions

We propose a new point-cloud segmentation network. It can effectively segment the crop-organ point cloud, and the average *Precision*, *Recall*, *F*1-*score*, and *IoU* of maize reached 99.35%, 99.26%, 99.30%, and 98.61%, while these metrics, for tomato, reached 97.98%, 97.92%, 97.95%, and 95.98%. At the same time, the time series is used as an input variable for training, which effectively improves the segmentation performance of the network. We further introduce an innovative sampling technique that enhances conventional FPS and VBS sampling methodologies. This method not only standardizes the number of points post-sampling but also circumvents overfitting resulting from sample imbalances. Such attributes render it particularly apt for the training and validation phases of deep learning frameworks. Based on semantic segmentation, the MeanShift algorithm is used to segment the crop point-cloud instance, and a higher segmentation accuracy is achieved. Finally, this article compares the evaluated network with several popular networks including PointNet, PointNet++, DGCNN, and JSNet.

Looking forward, we envisage potential refinements to the proposed technique by focusing on two main facets: firstly, our intention is to amass a more comprehensive collection of high-resolution crop point-cloud data and incorporate an extended array of species, with a particular emphasis on monocots characterized by elongated organs. Secondly, our endeavors will be directed towards architecting novel deep-learning frameworks tailored specifically for the nuanced comprehension and manipulation of 3D plant structures. Additionally, we aim to introduce compression networks that boast high segmentation precision, catering to the real-time requirements inherent to specific agricultural industry applications.

## Figures and Tables

**Table 1:** Accuracy evaluation of Point-cloud semantic segmentation.

	Maize		Tomato	
	Stem	Leaf	Stem	Leaf
*Precision* (%)	98.79	99.88	97.78	98.18
*Recall* (%)	99.90	98.59	98.47	97.36
*F*1-*score* (%)	99.33	99.23	98.12	97.78
*IoU* (%)	98.68	98.47	96.32	95.64

**Table 2:** Accuracy evaluation of point-cloud instance segmentation.

	Maize	Tomato
*Accuracy* (%)	98.45	96.12

**Table 3:** Quantitative comparison between our approach and other important semantic segmentation networks.

		Maize		Tomato	
		Stem	Leaf	Stem	Leaf
*Precision* (%)	PointNet	95.91	98.21	95.92	94.90
	PointNet++	96.49	97.80	97.34	96.87
	JSNet	95.80	95.33	95.72	96.73
	DGCNN	95.56	97.90	95.79	95.04
	Ours	98.80	99.89	96.98	97.80
*Recall* (%)	PointNet	97.64	94.51	94.45	92.86
	PointNet++	98.98	95.57	94.84	93.40
	JSNet	98.74	93.54	96.68	94.15
	DGCNN	99.89	92.20	96.50	94.61
	Ours	99.91	98.61	98.13	96.49
*F*1-*score* (%)	PointNet	96.77	96.32	95.18	93.88
	PointNet++	97.72	96.67	96.08	95.11
	JSNet	97.25	94.43	96.28	95.42
	DGCNN	97.67	94.96	96.15	94.82
	Ours	99.35	99.24	97.55	97.14
*IoU* (%)	PointNet	93.74	92.91	90.80	88.45
	PointNet++	95.54	93.56	92.45	90.67
	JSNet	94.65	89.45	92.84	91.25
	DGCNN	95.45	90.41	92.58	90.16
	Ours	98.71	98.50	95.22	94.44

**Table 4:** Ablation analysis on semantic segmentation.

	ASPP	Sampling	Date	Maize			Tomato		
				Stem	Leaf	Mean	Stem	Leaf	Mean
*Precision* (%)	√	√		98.80	99.89	99.35	96.98	97.80	97.39
	√		√	95.45	99.59	97.52	81.65	97.36	89.51
		√	√	98.00	99.70	98.85	94.36	91.66	93.01
	√	√	√	98.79	99.88	99.34	97.78	98.18	97.98
*Recall* (%)	√	√		99.91	98.61	99.26	98.13	96.49	97.31
	√		√	99.01	98.11	98.56	93.76	91.62	92.69
		√	√	99.74	97.67	98.71	92.56	93.69	93.13
	√	√	√	99.90	98.59	99.25	98.47	97.36	97.92
*F*1-*score* (%)	√	√		99.35	99.24	99.30	97.55	97.14	97.35
	√		√	97.20	98.85	98.03	87.29	94.40	90.85
		√	√	98.86	98.67	98.77	93.45	92.66	93.06
	√	√	√	99.33	99.23	99.28	98.12	97.78	97.95
*IoU* (%)	√	√		98.71	98.50	98.61	95.22	94.44	94.83
	√		√	94.55	97.72	96.14	77.44	89.40	83.42
		√	√	97.75	97.38	97.57	87.71	86.33	87.02
	√	√	√	98.68	98.47	98.58	96.32	95.64	95.98

## Data Availability

The data presented in this study are openly available in https://www.ipb.uni-bonn.de/data/pheno4d/ (accessed on 15 November 2023).
